# Soil water dynamics and biomass production of young rooibos (*Aspalathus linearis*) plants

**DOI:** 10.1038/s41598-023-41666-5

**Published:** 2023-09-13

**Authors:** Roeline van Schalkwyk, J. Eduard Hoffman, Ailsa G. Hardie, Johan L. van Zyl

**Affiliations:** Department of Soil Science, Stellenbosch University, Stellenbosch, South Africa

**Keywords:** Plant physiology, Climate sciences, Environmental sciences, Hydrology

## Abstract

Rooibos (*Aspalathus linearis*) is endemic to certain regions of the Western- and Northern Cape of South Africa, where it is also commercially grown. Being low-rainfall regions, information on the soil water balance of rooibos is essential, but such data is limited. Consequently, the effect of inorganic fertilisation and soil depth on soil water dynamics in a young rooibos plantation at Nardouwsberg, Western Cape were studied. Soil water content of plots planted to unfertilised and fertilised plants as well as that of bare soil were determined over the duration of the 2016/17 season. All treatments were replicated on shallow and deep soils sites and plant growth was determined at the end of the season. At the end of the study, the profile soil water content and evapotranspiration of the bare and planted plots were similar which prove that fallowing (water harvesting) is not an option in the sandy soils of this region. With the exception of the 20−30 cm root zone of the planted plots at the deep site, the water content decreased to levels below the permanent wilting point in the soil profile during summer. It was concluded that rooibos plants could survive through an adapted root system. A further survival method was proposed, involving moisture moved through evaporation from the deeper soil layers into the drying-front in the ~ 10−30 cm soil layer where a condensation-evaporation cycle enables rooibos to harvest small amounts of water. The highest shoot biomass with the longest taproot resulted from the unfertilised treatment on the deep soil thanks to higher soil water content, whereas the shoot and root biomass of the fertilised treatment at both sites were low due to high P soil concentration. This study revealed that unfertilised plants on deeper soils result in higher rooibos production under drought conditions.

## Introduction

The rooibos plant (*Aspalathus linearis*) is a leguminous shrub^1^, belonging to the Fabaceae family^2^, and grows in the Mediterranean climate^3^ of the Cape Floristic Region of the Western- and Northern Cape provinces of South Africa^4^. Although, this special plant had no commercial interest at the beginning of the twentieth century, the cultivated area reached 95 000 ha in 2016^5^. Over the last ~ 18 years, the production of rooibos has varied between 10,000 and 18,000 tons per year, all of which occurred under dryland production^6^. Since its commercialisation, there has been a greater research focus on the health benefits and quality of rooibos tea than on the specific soils where rooibos likes to grow. It was reported by Stassen^7^, who did research on soil properties related to rooibos cultivation, that rooibos prefers soils which are deep and cooler with higher soil water storage (SWS).

The negative effect of temporary seasonal drought can be mitigated by fallowing, a practice that can increase stored soil water by conserving rainfall. Fallow efficiency (FE) is improved, amongst other factors, by deeper soils and a higher amount of plant residue on the soil surface^8^. Fallowing is mostly practised in areas and for crops where rainfall precedes, the planting of a new crop e.g. winter wheat in a summer rainfall area^9^. Fallow efficiency after long dry summers is, however, still unknown in the case of rooibos that is normally planted during winter, between June and August.

Soil evaporation (E) in semi-arid or arid regions causes the greatest loss of water^10^ and the evaporation demand is usually greater than the ability of soil to conduct water in the liquid phase^11,12^. Several researchers noted that the loss of water due to evaporation from bare soil is between 50 to 70% of the annual rainfall^13–15^. Due to the scarcity of water resources, knowledge about water liquid and vapour movement regarding the developing and extending of dryland farming, has gained importance. A number of researchers have studied the phenomenon of soil drying ^16–18^, however, during past years, it has been recognised that water vapour flow plays a crucial role by transporting water and vapour of chemicals. Furthermore, the water vapour flow might be important in maintaining plant growth and healthy ecosystems^19–21^. Soil evaporative drying occurrs in three phases^22,23^: (I) the initial constant-rate, (II) the intermediate falling-rate, and (III) the residual slow-rate. During the initial constant-rate phase, evaporation will increase rapidly in the soil surface after a sufficient rainfall and is limited to atmospheric evaporative demand (AED). As the soil drying process continues, the water availability in soil surface is decreased with a lower evaporation rate below the potential rate, the falling-rate phase will begin. The residual slow-rate is established after the soil surface has become so desiccated that the conductance of liquid water ceases and this phase can persist for a long period. During the residual slow-rate of the soil evaporative drying, the location of the evaporation zone in the soil surface shifts from the surface to the subsurface, resulting in formation of a (5−50 cm) drying-front^22,24,25^/dry soil layer^26–28^. There is a sharp transition from the Phase I to the Phase II, but the transition between Phase II & III occurs gradually^29^. Therefore, Hillel^22^ proposed that it is the best to describe the evaporation process in two stages: (I) initial constant-rate stage, and (II) falling-rate stage where Phase II & III seen as one stage.

A study was consequently undertaken to investigate the soil water balance (flow of water in and out of the soil profile) of rooibos production. The first aim of this investigation was to determine the effect of fertilisation, soil depth and fallowing on the soil water balance of selected rooibos plants from seedling stage to one-year old mature plants. The second aim was to investigate root development and distribution, and to link it with above-ground growth and soil water availability.

## Materials and methods

### Experimental layout

The study was conducted at Nardouwsberg, on Vaalkrans farm (32°00′38.2″S, 18°55′19.0″E; 570 m mean height above sea level) 20 km northwest of Clanwilliam, Western Cape. This region has a typical Mediterranean climate with warm, dry summers and mild, wet and cold winters^30^. In this climatic region, 90% of the annual rainfall occurs during winter (June to August)^31,32^. The optimum annual rainfall for rooibos production is at least 300–350 mm^33^, but global climatic models estimate^34^ that the total winter rainfall in the rooibos production region will decrease to below 165 mm annually^33^. The 2016/17 season was particular a dry year with only 98.9 mm of rain, where the maximum daily air temperature reached 47.49 °C in the summer and 6.44 °C in winter, respectively. The soil in the study area is a sandstone-derived sand that is sometimes underlain by hard or soft relic plinthic material, and belongs to the Cartref soil form according to the South African Soil Taxonomy System^35^.

**Figure 1:**
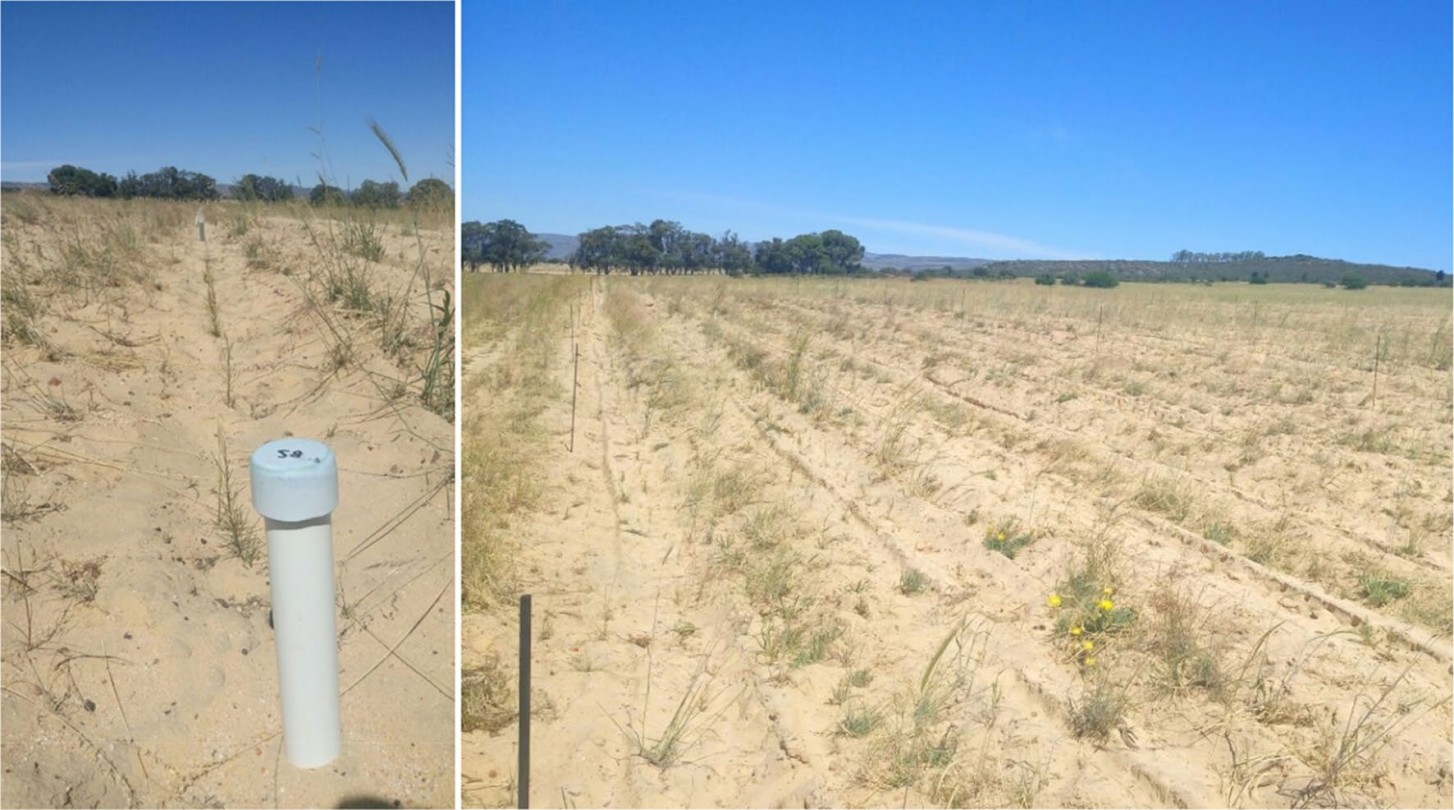
Rooibos plantations with the polyvinyl chloride access tubes for the Diviner 2000 soil water content readings (photos taken on 4 October 2016).

The field trial commenced on 16 June 2016 when the fertilisers were applied by hand to the planting rows and the five-month-old rooibos seedlings (sown in February 2016) were planted (Fig. 1). The cultivation of plants in the present study complies with national and institutional guidelines. Three treatments were applied on both the shallow (50 cm) and deep (100 cm) soils i.e. a total of six treatment combinations and the layout on each soil was a randomised block design with four replications. The three treatments were: (1) unfertilised planted soil, (2) planted soil receiving moderate amounts of nitrogen (13.8 kg ha^-1^), phosphorous (21 kg ha^-1^) and potassium fertiliser (14 kg ha^-1^) and (3) bare, unplanted soil. Each plot of the planted treatments consisted of 6 rows of 12 rooibos plants (bushes planted 0.75 m apart) with a row spacing of 1.5 m wide. The total plot area was 81 m^2^. The soil at both sites was homogenous and contained 91–98% sand, 0.89–8.85% silt and 0.10–2.20% clay. The bulk density of this soil at both sites was 1.65 g cm^-3^ with field capacity of 92 mm m^-1^ (at -9 kPa), permanent wilting point (PWP) of 17 mm m^-1^ (at − 1500 kPa) and a 75 mm m^-1^ water holding capacity (WHC).

### Soil water content, evapotranspiration and soil evaporation

The soil water content (SWC) was measured with a capacitance probe (Diviner 2000, Sentek Sensor Technologies Inc, Stepney, Australia). A polyvinyl chloride (PVC) access tube was installed *ca*. 15 cm from a rooibos plant within the row on each replicate plot of the unfertilised and fertilised treatments on both soil sites. For each bare treatment plot, the PVC access tube was installed in the middle between the third and fourth row. Using a hand auger, both soils were augered to the bedrock which occurred at 105 cm in the deep soils and 50 cm in the shallow soils. The PVC access tubes were then driven into the augered holes leaving *ca.* 10 cm of the tubes above ground. The SWC was measured in 10 cm increments down to 30 cm depth for the shallow soils and 80 cm depth for the deep soils. Measurements were taken weekly during the growing season from July 2016 to April 2017. A soil-specific calibration of the Diviner 2000 was done gravimetrically in the field and the calibration equation for Diviner 2000 was:

1 $$ {\text{VWC}}_{{{\text{calibrated}}}} = {1}.0{43} \times {\text{VWC}}_{{{\text{field}}}} {-}0.0{38} $$

where VWC_calibrated_ is the calibrated volumetric water content (m^3^ m^-3^) and VWC_field_ is the volumetric water content by the Diviner 2000 in the field (m^3^ m^-3^) with a coefficient of determination was 0.76 and the root mean square error was 0.003 m^3^ m^-3^.

Management of unwanted water losses and maximising of water storage is critically important in dryland crop production. The soil water balance equation of Hillel^22^ is essential to calculate such soil water gains and losses. Stewart and Peterson^36^ implied, however, that only the change in soil water content, precipitation and evapotranspiration parameters are important in dryland farming. This was also true of the current experiment since the upward capillary flow (there was no water table/free water), runoff and drainage (soil water contents were below field capacity of each soil layer) were negligible. Lu et al.^37^ reported similar approach for sandy soils under dryland farming of winter wheat. The evapotranspiration (or evaporation in the case of bare soil) was therefore calculated for each treatment by using the following Eq.^37^:

2 $$ {\text{ET }} = \Delta {\text{SWC }} + {\text{ P}} $$

where ET is evapotranspiration (mm), ΔSWC is change in soil water content (mm) and P is precipitation (mm).

The cumulative evapotranspiration was also calculated by adding the weekly evapotranspiration over the duration of the growing season as follows^38^:

3 $$ \sum {\text{ET = ET}}_{{\text{i}}} {\text{ + ET}}_{{\text{i + 1}}} { + } \cdots {\text{ + ET}}_{{\text{n}}} $$

where ΣET is cumulative evapotranspiration (mm), ET_i_ is evapotranspiration of week i (mm), ET_i+1_ is evapotranspiration of the following week (mm) and ET_n_ is evapotranspiration of the nth week (mm).

The fallow efficiency on the bare treatment at shallow and deep sites was determined, using Eq. 4^39^:

4 $$ {\text{FE}} = \frac{{\Delta {\text{SWC}}}}{{\text{P}}} \times 100 $$

where FE is fallow efficiency (%), ΔSWC is the change in the soil water content (mm) and ΣP is cumulative precipitation (mm).

In addition, ECH_2_O sensors (Meter Group Inc., Pullman, Washington, USA) were installed in unfertilised, fertilised and bare experimental blocks at the deep site to measure the soil temperature and water content. The procedures of ECH_2_O sensors are further explained in the following section.

### Drying front and hydraulic diffusivity

Four ECH_2_O sensors (5TM, EC-20, EC-TM and GS1) were installed in the unfertilised, fertilised and bare experimental block on the deep soil to measure the VWC. These ECH_2_O sensors were installed horizontally in the soil profile *ca.* 15 cm from the PVC access tubes of the Diviner 2000 at 5, 15, 25, and 50 cm soil depths. These sensors from each treatment were directly connected to an ECH_2_O datalogger^40^. The datalogger was programmed to measure the VWC and soil temperature every 10 min. Measurements of the volumetric water content (VWC) were conducted from July 2016 to September 2017. A soil-specific calibration of the ECH_2_O sensors was done in the laboratory as described by Cobos and Chambers^41^. The calibration equation for the sensors was:

5 $$ {\text{VWC}}_{{{\text{calibrated}}}} = \, 0.{57}0 \times {\text{VWC}}_{{{\text{field}}}} {-} \, 0.0{17} $$

where VWC_calibrated_ is the calibrated volumetric water content (m^3^ m^-3^) and VWC_field_ is the volumetric water content measured by the sensors in the field (m^3^ m^-3^) where the coefficient of determination was 0.86 and the root mean square error 0.008 m^3^ m^-3^.

The sink strength of the rooibos plants was not accounted in Eqs. 6−7 (plants were still immature). There are several soil water diffusivity models, however, the model of Doering^42^ and Black et al.^43^ was used to calculate the diffusivity coefficient. Black et al.^43^ changed Gardner & Hillel model^44^ (Eq. 7) and rewrote Eq. 6 by replaced the soil evaporation rate with d*θ*/dt to determine the diffusivity coefficient directly. The diffusivity coefficients of the soil profile up to 25 cm and in the three soil layers; 5, 15 and 25 cm; were determined by using the VWC data as follows^42,43^:

6 $$ {\text{D}}\left(\theta \right){\text{ = 4L}}^{{2}} \frac{{{\text{d}}\theta {\text{/dt}}}}{{\uppi ^{{2}} {(}{\theta  - \theta }_{{\text{f}}} {)}}} $$

where D(*θ*) is diffusivity coefficient (mm^2^ day^-1^), L is length of profile (mm), d*θ*/dt is instantaneous rate of water loss (mm^3^ mm^-3^ per day), *θ* is instantaneous volumetric water content (mm^3^ mm^-3^) and *θ*_f_ is final volumetric water content (mm^3^ mm^-3^). Parlance et al.^45^ found that the simplified model of Doering (1965) and Black et al. (1968) can applied in field scale experiments to obtain the diffusivity coefficients. Hoffman^46^ further evaluated the hydraulic diffusivity model (Eq. 6) and proved that the diffusivity coefficients of the soil layers can be determined. By doing so, Hoffman^46^ conducted lysimeter experiments (6 cm in diameter with a height of 31.5 cm) and measured the evaporation rate at 0−10, 10−20 and 20−30 cm soil layers. Hoffman^46^ determined the average diffusivity coefficient of the three soil layers and used the diffusivity coefficients in Gardner and Hillel (1962) model (Eq. 7). Thereafter, the measured and the simulated evaporation rates were compared, and the validation was satisfactory^46^. The soil evaporation rate, *e*, was calculated by the following equation of Gardner & Hillel^44^:

7 $$ e = - \frac{{{\text{dE}}}}{{{\text{dt}}}} = \frac{{{\text{D}}\left( {\theta _{{{\text{ave}}}} } \right){\text{W}}\uppi ^{2} }}{{4{\text{L}}^{2} }} $$

where D(*θ*_ave_) is the diffusivity coefficient at the average water content of a soil profile (mm^2^ day^-1^), W is total amount of water in the soil profile (mm) and L is length of the profile (mm). The thermal flux of *Fourier’s law* was also calculated:

8 $$ q_{{\text{h}}} = \, - \kappa {\text{T}} $$

where *q*_h_ is the thermal flux (W m^-2^), κ is the material’s conductivity (W m^-1^ K^-1^) and $\nabla$ T is the spatial temperature gradient (K m^-1^).

### Above and below ground biomass, and taproot length

One rooibos tea plant was destructively harvested by hand on each of the four planted treatments on 22 February 2017, 26 May 2017 and 27 September 2017. Harvesting was done by hand using a pruner and shovel to determine above and below ground biomass separately. The roots were washed out with potable water on top of a 0.053 mm sieve to prevent fine roots from being lost. Following this, the shoots and roots were oven dried at 60 °C until no mass losses occurred. Above and below ground biomass were expressed as total mass (g) per plant. The length of the taproots was measured manually using a cotton rope and metal ruler. The root masses were determined at 0–10, 10–20, 20–30, 30–40 and > 40 cm soil depth increments. Care was taken to harvest the whole root system of every sample plant.

### Statistical analysis

An appropriate analysis of variance (ANOVA) was performed, and the data was tested for significant statistical differences with 95% confidence interval.

## Results

### Soil water content

**Figure 2:**
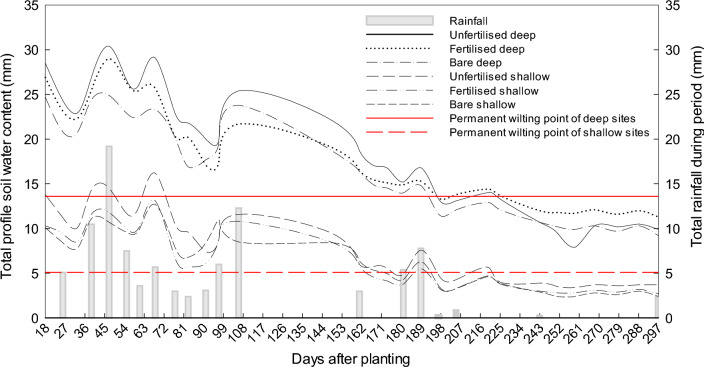
Average total profile soil water content for the unfertilised, fertilised and bare treatments on deep and shallow soils during the 2016/17 growing season.

During the study period, the rainfall was not sufficient to fill either the shallow or the deep soil to field capacity. The highest soil water content was measured on 23 August in the shallow soil and a bit earlier, namely on 2 August in the deep soil irrespective of whether they were fertilised or not. The deep soil had a much higher SWC than the shallow soils throughout the investigation (Fig. 2). In the winter season (July–August 2016), the total SWC in the shallow and deep soils (both unfertilised and fertilised treatments included) ranged between 8.6–14.5 mm (64.4% of the WHC) and 22.4–30.4 mm (50.7% of the WHC), respectively (Fig. 2). During the summer season (November 2016 to February 2017), the total SWC of the shallow soils ranged between 2.8–5.7 mm versus 11.7–16.8 mm in their deep counterparts. Notably, the profile SWC of the planted treatments on the shallow soil reached PWP much earlier in the season than their counterparts on the deep soil, namely (Fig. 2):

- Shallow soil—Unfertilised rooibos plants: 7/12 (174 days after planting) and fertilised rooibos plants: 30/11 (168 days after planting); and
- Deep soil—Unfertilised rooibos plants: 28/01 (225 days after planting) and fertilised rooibos plants: 28/01 (225 days after planting).

**Figure 3:**
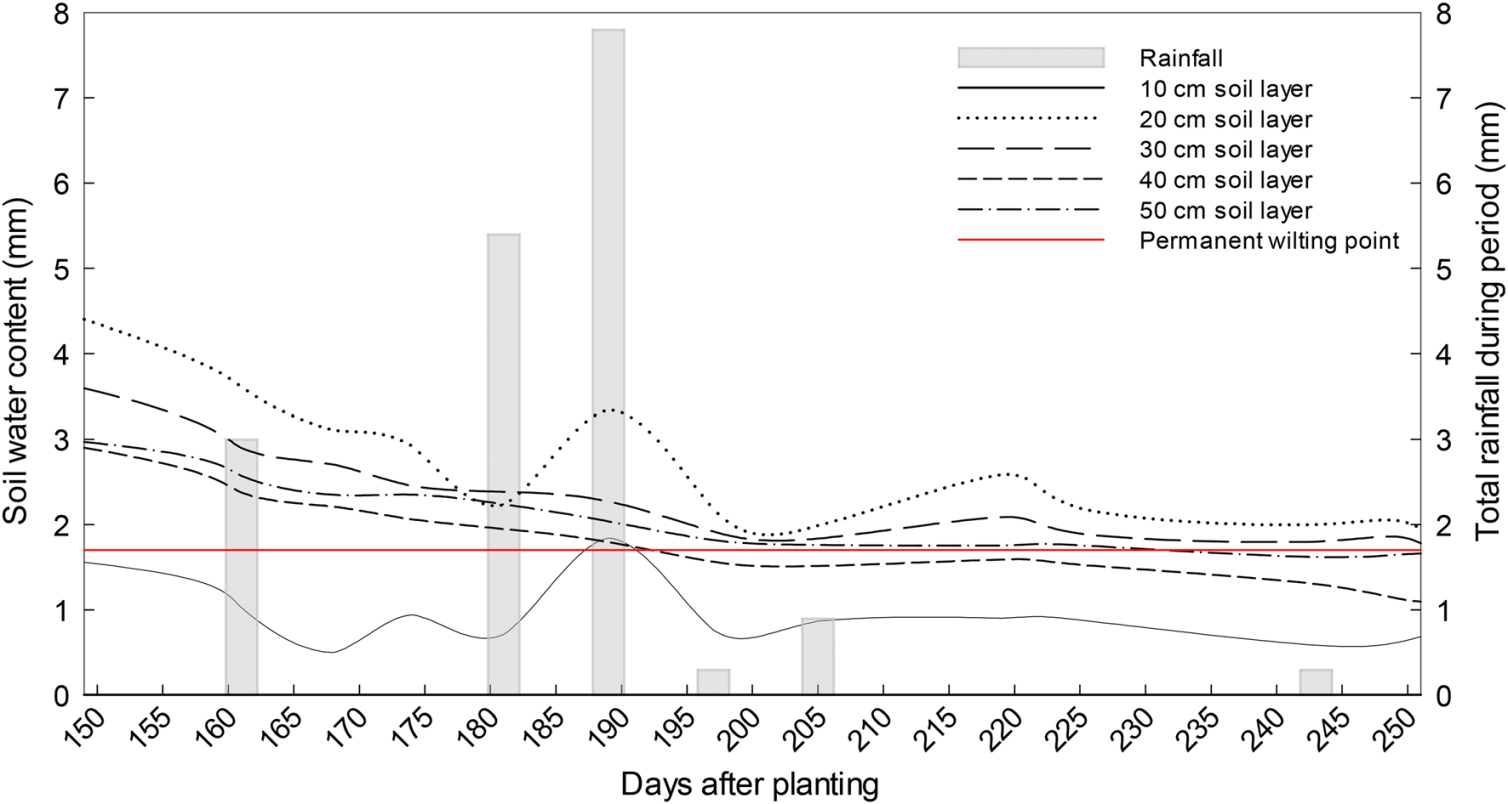
The soil water content of unfertilised treatment at deep site during summer (November−February). The red line indicate the permanent wilting point at 1.7 mm.

Interestingly, the SWC at 20 and 30 cm soil layer of the unfertilised treatment at the deep sites did not fall below PWP during summer (Fig. 3). The fertilised treatment at the deep sites showed a similar trend, whereas both planted treatments at the shallow sites dried out below PWP. The SWC differences between fertilised and unfertilised treatments were small. At the deep site, for the first part of the season, the unfertilised treatment had a slightly higher SWC than the fertilised treatment. This difference reached a maximum of 3.7 mm at 106 days after planting. From day 197 onwards the order switched around and at the end of the season the fertilised treatment stored 1.4 mm more water than the unfertilised treatment, but this difference was not significant (P = 0.06). In the shallow soils, the largest significant difference (P = 0.01) between the two treatments occurred at the beginning of the season and reached a maximum of 3.9 mm at 83 days after planting. For the largest part of the season, however, i.e. between day 97 and 297 difference in SWC between the two treatments were small and non-significant (P = 0.26).

In general, the SWC of the two bare soils closely followed the depletion and replenishment patterns of their planted counterparts. In fact, despite the absence of water extraction by plants, no cumulative storage of rainwater on bare soils is evident from the data. A comparison of the two bare soil treatments showed that on the deep soils, the total SWC ranged between 9.2–24.9 mm and from 2.4 to 12.7 mm on the shallow soils (Fig. 2). At the end of the season, the deep soil contained 6.8 mm more water than the shallow soil (P < 0.05).

### Evapotranspiration and soil evaporation

**Figure 4:**
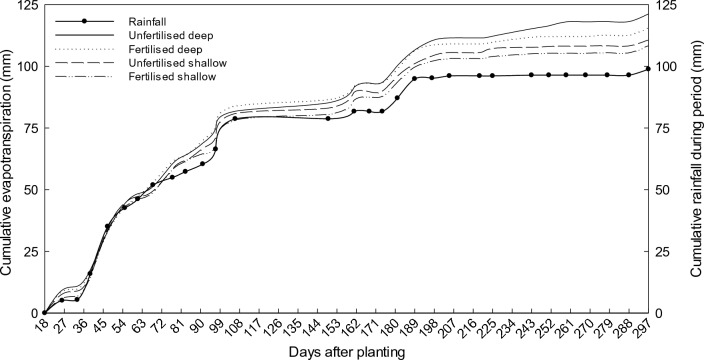
Average cumulative evapotranspiration for the unfertilised and fertilised treatments on deep and shallow soils during the 2016/17 growing season.

Rainfall was only sufficient up till 47 days after planting when it was higher than the cumulative evapotranspiration (ΣET) for the two treatments on both sites (Fig. 4). The ΣET of the fertilised and unfertilised treatments on both sites increased much more rapidly in the early part of the season i.e. from planting until day 106 (30 September), than during the rest of the season (Fig. 4). In fact, the four treatments lost on average 81.3 mm water during the first 106 days and only 32.7 mm during the rest of the season from 30 September–11 April (190 days). It is clear that the high evapotranspiration (ET) rate coincided with the 78.7 mm of rain that fell during the early part of the season in this winter rainfall region. The decrease in ET rate after 30 September occurred despite an increase in leaf area of the rooibos plants and high summer temperatures. The reduced SWC led to the ΣET reaching a nearly steady rate until effective rainfall events occurred from 161 to 189 days after planting. After 189 days following planting, the ΣET again reached a nearly steady rate and this continued until a significant rainfall occurred to supply sufficient water for the evaporative demand.

A summary of the soil water balance (SWB) for the six treatments indicated, as expected, that all the soils were drier at the end of the season than at the first day of planting (Table 1). Stored water from the deep soils contributed an average of 17.1 mm to the water use of the rooibos plants while this figure was only 9.6 mm for the shallow soils. The total water use (ƩET), varying from 108.4 mm to 121.2 mm was not much different among the four planted treatments (P = 0.88). Perusal of the 2016/17 water balance on the two bare soil treatments showed a slightly higher cumulative evaporation (ƩE) of 7.5 mm on the deep soil (Table 1). Unexpectedly, within each group of soil depth treatments, ƩE of bare soil was almost similar to ƩET of soil planted to rooibos (P = 0.96).

**Table 1:** Summary of the average soil water balances and the fallow efficiency of the unfertilised, fertilised and bare treatments at shallow and deep sites during 2016/17.

Treatment	Site	SWC–start	SWC–end	ΔSWC	ΣΕΤ	ΣΕ	FE
		mm	mm	mm	mm	mm	%
Unfertilised	Deep	28.5 (*1.20*)	9.9 (*0.55*)	18.6 (*0.99*)	121.2 (*0.98*)	-	-
Fertilised		26.9 (*1.95*)	11.3 (*0.66*)	15.6 (*2.23*)	110.7 (*2.14*)	-	-
Bare		24.7 (*1.97*)	9.2 (*0.50*)	13.5 (*1.67*)	-	116.1 (*2.11*)	-(14.05)
Unfertilised	Shallow	13.8 (*0.36*)	3.7 (*0.31*)	11.1 (*0.05*)	115.8 (*0.05*)	-	-
Fertilised		10.3 (*0.31*)	2.7 (*0.21*)	7.6 (*0.34*)	108.4 (*1.48*)	-	-
Bare		10.1 (*0.29*)	2.4 (*0.13*)	7.7 (*0.38*)	-	108.6 (*0.40*)	-(8.01)

SWC-start = soil water content at the beginning of season 2016/17, SWC-end = soil water content at the end of season 2016/17, ΔSWC = change in soil water content (ΔSWC = SWC-start – SWC-end), ΣET = cumulative evapotranspiration, ΣE = cumulative evaporation, FE = fallow efficiency.

The standard errors are in parentheses.

### Drying front and hydraulic diffusivity

**Figure 5:**
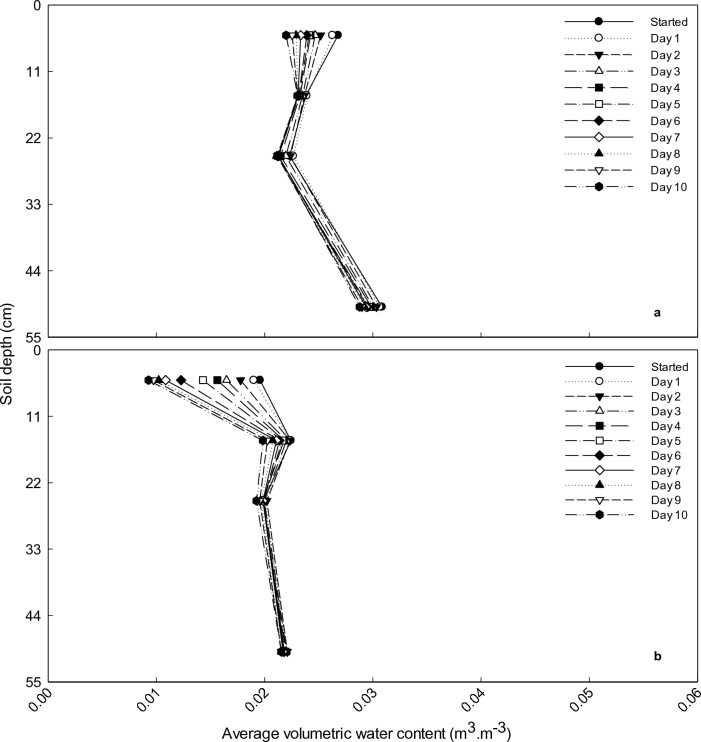
Development of a drying front on 1–11 November 2016 (**a**) and on 3–13 January 2017 (**b**) during in the summer in the deep soils (unfertilised plants).

**Figure 6:**
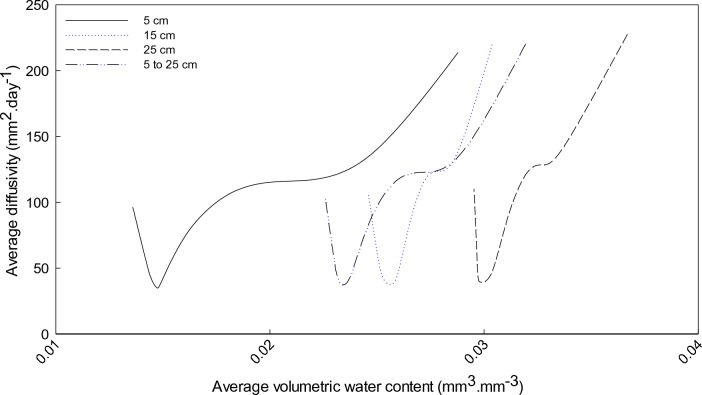
Average diffusivity coefficients at 5, 15 and 25 cm soil depths on 17–25 September 2016 of unfertilised plants at the deep site.

The ability of rooibos plants to survive and produce a crop under conditions of extremely low rainfall suggests an effective adaptation to its environment. In addition to a two-tier root system and above-ground plant morphological factors, it has been suspected that rooibos plants can also access moisture, e.g. condensed water in the soil, other than soil water normally determined. With this hypothesis in mind, drying front and diffusivity were determined for all treatments, however, only the drying front and diffusivity of the unfertilised treatment on the deep soil were displayed in Figs. 5, 6. A drying front was observed between 15 and 25 cm in Fig. 5a and b at 0.02 and 0.03 m^3^ m^-3^. Moreover, the drying front can occur at a VWC less than 0.02 m^3^ m^-3^ in this specific soil (data not shown). These results showed that the drying front in the rooibos soil was ~ 10−30 cm thick. The 5 cm soil layer had the lowest VWC where the vapour diffusivity started at VWC of 0.02 m^3^ m^-3^ (Fig. 6). Vapour diffusivity of the 15 and 25 cm soil layer commenced at an average VWC of 0.034 and 0.026 m^3^ m^-3^. Based on the hooked form of the diffusivity vs. water content function^47^, the average vapour diffusivity for 5–25 cm and 15–25 cm soil layers started at VWC of 0.025 and 0.03 m^3^ m^-3^ (Fig. 6), similar to the VWC < 0.06 m^3^ m^-3^ in a study done by Philip^48^.

**Figure 7:**
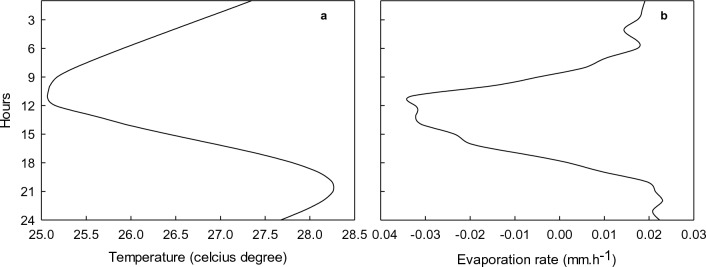
Average soil temperature (**a**) and evaporation rate (**b**) in the drying front (~ 10−30 cm in soil of the experiment) for unfertilised plants at the deep sites during the summer season.

In Fig. 7a and b illustrated the average soil temperature and drying-wetting cycle in the unfertilised plant soil profile at the deep site, respectively. Note that the negative E rate represented vapour condensation (Fig. 7b). The maximum of the average E rate was *ca.* 0.02 mm h^-1^ during early morning and late evening. The condensation took place when the soil temperature was at the lowest (25.07°C) and with an average E rate of *ca.* -0.03 mm h^-1^. The quantities of water that can condense are small and will be an average of 0.33 mm day^-1^ (0.01−1.30 mm day^-1^) when using Fig. 5b for calculation purposes. If it is assumed that each rooibos plant exploits an area of 0.75 m × 0.75 m (0.75 × 1.50 m planting distance), the volume of water that can be harvested through condensation in the drying front zone will be 185.6 ml day^-1^ (56.3−731.3 ml day^-1^).

### Above- and below-ground biomass, and taproot length

**Figure 8:**
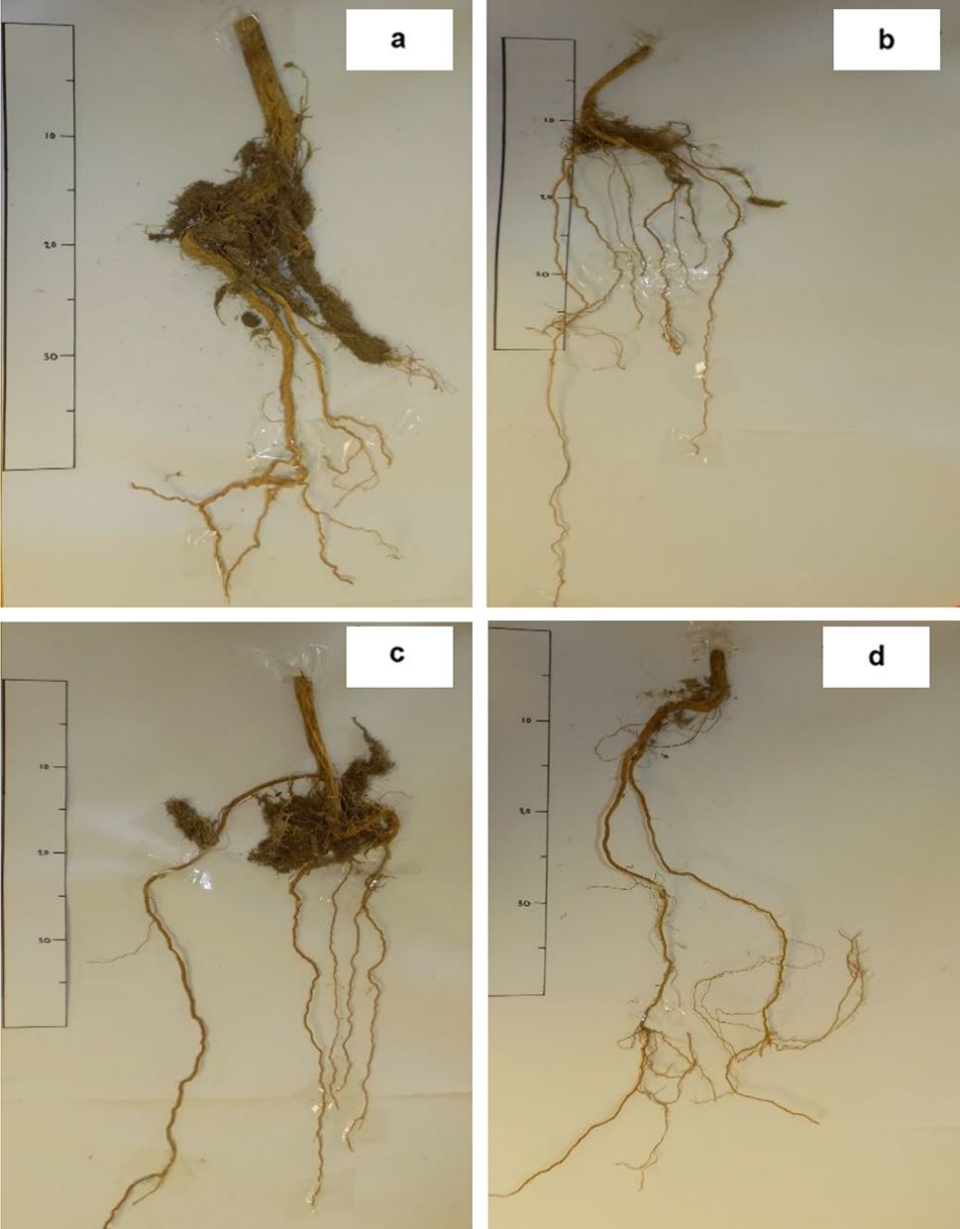
Root morphology of rooibos plants of the unfertilised (**a**) and fertilised (**b**) treatments at the shallow site. The unfertilised and fertilised treatments at the deep site is (**c**) and (**d**), respectively.

On 25 September 2017, approximately 15 months after planting the above-ground biomass (AGB) of the unfertilised deep soil was significantly higher than its shallow counterpart (Table 2), most probably due to higher SWC as discussed previously. Total below-ground biomass (BGB) followed a similar pattern on the unfertilised treatment (Table 2). Noteworthy is the fact that the AGB:BGB ratio was exactly the same (0.28) for the two unfertilised treatments irrespective of soil depth which implies that the AGB and BGB of the rooibos plants were in balance. Furthermore, the cluster roots grew in the ~ 10−30 cm soil depth of all the planted treatments (Fig. 8).

**Table 2:** Summary of above ground biomass and below ground biomass on 25 September 2017 and taproot length at different soil depths (cm) of unfertilised and fertilised treatments at deep and shallow sites.

Treatment	Site	AGB	BGB						Taproot length	
			0–10 cm	10–20 cm	20–30 cm	30–40 cm	> 40 cm	Total	22 Feb. 2017	26 May 2017
Unfertilised	Deep	173.85^a^	21.49	12.45	2.47	2.99	7.77	47.17^a^	33.32^a^	70.67^a^
Fertilised		56.60^c^	7.97	3.68	2.38	1.63	2.46	18.12^c^	27.49^b^	63.83^b^
Unfertilised	Shallow	147.47^b^	19.28	11.83	2.58	6.72	-	40.68^b^	36.58^a^	65.13^b^
Fertilised		17.57^d^	6.65	4.44	0.79	3.76	-	15.46^c^	35.78^a^	55.05^c^

AGB = above ground biomass (g), BGB = below ground biomass (g), taproot length is in cm.

Different superscript within each column indicates significant difference (P < 0.05).

The taproot lengths of all treatments were similar on 22 February 2017 (Table 2). This implied that the rooibos plants were still immature at that stage i.e. about eight months after planting. During the next three months (22 February to 25 May), however, the taproots extended at different rates and ended up significant longer on the deep soil than on the matching shallow soil treatments.

## Discussions

The comparison between deep and shallow soil, clearly demonstrated the benefit of deep soil on soil water conditions and in turn, also on plant performance. Irrespective of soil depth, for the largest part of the season, the unfertilised treatment plots had a higher soil water content than fertilised ones. This result may be due to (1) higher percentage surface cover which was observed in a parallel study on the same rooibos planting during the 2016/17^49^ and (2) soil temperatures that were markedly lower at both 5 and 15 cm soil depths compared to the fertilised treatment (Table 3). In the current study, rooibos plant growth did not respond positively to fertilisation, but instead most of the fertilised plants grew poorly^49^. This observation is supported by the research of Harris^50^ who reported decreased growth of *Protea obtusifolia* from 8.1 to 7.8 cm when P increased from 1 to 10 mg kg^-1^ in the Cape Floristic Region. Corbella-Tena et al.^51^ also reported that the growth of *Leucospermum cordifolium* ‘Flame Spike’ decreased during the different growth stages (6, 9 and 12 months) if the P concentration increased. Moreover, a P toxicity effect was found on the young plants if the concentration was higher than 5 mg kg^-1^ in the aforementioned study. Therefore, a smaller percentage surface cover of the fertilised rooibos plants in the current study caused more direct sunlight on the soil surface and the soil temperature increased. The average maximum soil temperature of the fertilised treatment in the 0–10 cm soil layer was significantly higher (P < 0.05) by 3.86°C than in the unfertilised treatment (Table 3). In the 10–20 cm soil layer, this difference in soil temperature was only 1.5°C (Table 3) which was not significant (P = 0.08) due to delayed energy transfer. The difference in the SWC between deep and shallow bare soils is, however, meaningless for the potential production of rooibos during the following growing season since all soil layers on both soils were already far below PWP at this stage.

**Table 3:** Mean maximum soil temperature (T_soil_ in °C) in the unfertilised and fertilised treatment of the deep soils at 5 and 15 cm soil depths.

Days after planting	Unfertilised	Fertilised	Difference in maximum T_soil_	Unfertilised	Fertilised	Difference in maximum T_soil_
	Maximum T_soil_ at 5 cm	Maximum T_soil_ at 5 cm		Maximum T_soil_ at 15 cm	Maximum T_soil_ at 15 cm	
149	28.9	34.6	5.7	26.8	28.2	1.4
161	25.4	28.5	3.1	24.7	25.9	1.2
168	30.2	34.7	4.5	28.4	29.8	1.4
174	30.7	36.7	6.0	28.4	29.7	1.3
181	30.6	35.2	4.6	28.9	30.0	1.1
189	27.2	29.9	2.7	25.9	26.4	0.5
197	33.5	36.3	2.8	31.7	33.3	1.6
205	34.2	39.7	5.5	31.6	33.2	1.6
219	34.0	38.7	4.7	30.2	31.9	1.7
225	31.2	35.6	4.4	27.7	29.3	1.6
243	34.6	38.5	3.9	29.8	32.0	2.2
251	35.1	38.4	3.3	30.6	32.9	2.3
259	29.0	30.8	1.8	27.8	28.3	0.5
268	32.8	36.3	3.5	28.6	30.4	1.8
278	34.7	37.9	3.2	30.1	32.2	2.1
288	29.9	31.8	2.9	25.8	27.1	1.3
279	27.6	30.7	3.1	24.7	25.9	1.2

The limited ET in the summer season is linked to reduced SWC and lower rainfall^52,53^. According to Gardner^54^ and Gardner et al.^55^, the ET rate is higher from a wetted soil than from a drier soil. Therefore, there was a sharp increase in ΣET after rainfall events (Fig. 3). For dryland farming in Italy (Mediterranean region), the daily ET showed an increase from 2.1 mm day^-1^ to a value ranging between 2.39 and 2.87 mm day^-1^ after a rainfall event above 10 mm^56^. Supporting evidence is provided by a South African study in the Renosterveld vegetation of the Voëlvlei Nature Reserve where the ΣET of 682 mm was higher in the winter season than the total summer evapotranspiration of 620 mm^57^. The ƩET value was only slightly higher for the two unfertilised treatments than for the fertilised plots. Soil depth had also no obvious effect on ƩET over the full season.

The higher ƩE of the deep soil is probably linked to the higher SWC in the deep soils. The SWC of bare soils on both shallow and deep sites was below their respective PWP’s by the end of the season. Therefore, fallowing does not seem to be a feasible option for the establishment of rooibos plants in this dry region. This conclusion is underlined by the negative FE values on both the shallow and deep soil.

Overall, the diffusivity coefficient (35.02–236.11 mm^2^ day^-1^) is lower compared to 961 mm^2^ day^-1^ reported by Black et al.^43^ for sandy soils. Parlance et al.^45^ mentioned that the field hydraulic diffusivity will be lower than the laboratory hydraulic diffusivity and also the low values may be due to the fact that the soils in the current study were dry. Similar low values of 45–432 mm^2^ day^-1^ for sandy loam soils in the Free State Province, South Africa was reported by Hoffman^46^.

The soil water content and hydraulic diffusivity results in the current study, suggested that the water movement in the rooibos soil occurred through vapour diffusivity due to drought with large temperature gradients most of the time. Kuzmak & Sereda^58^; Matthes & Bowen^59^, and Jabro^60^ reported that the water flow response to the thermal gradient through unsaturated soils is mainly in vapour phase under arid and semiarid regions. Vapour flow accounts for ~ 30% of the total water flux under dry soil conditions, where the vapour and liquid fluxes can be in the same order of magnitude^61,62^. In addition, the water transport mechanism in dry and coarse-textured soils is mainly vapour flux associated with the evaporation–condensation process (drying-wetting cycles)^63–65^. Jackson^63^ further concluded that the vapour phase can be an important transport vehicle for water and chemicals in dry soils. It can therefore be suggested that another likely survival factor of the rooibos plants can be their dependence on the drying-wetting cycles in a drying front scenario. Depending on solar radiation and soil temperature, a drying front can in either vapour or liquid phase^66^. A drying front can act as an evaporation (E) zone between late morning and early afternoon and as a condensation zone when soil temperature is lower^26^.

In the current study, the evaporation occurred during early morning and late evening. According to the Philip and De Vries theory^67^ for describing the liquid-heat-vapour dynamic processes, there are three phases (i.e., Phase I to III) of the liquid-heat-vapour dynamic processes in porous materials (soils included). In Phase I, the soil is sufficiently wet and evaporation is indistinguishable from a saturated surface (0−5 cm), whereas in Phase III, the soil is dry and E may negatively correlate with the thermal flux. During Phase II & III, evaporation takes place within the soil mass (e.g., in the E zone below the drying front) and the vapour diffuses upward through the drying front (wetting and drying cycle with condensation/re-evaporation can occur here) to the surface and then into the atmosphere^67^. Since the rooibos soils were dry in the current study, the liquid-heat-vapour dynamic processes could have been in Phase II & III (or the falling-rate stage). Furthermore, the Pearson correlation coefficient (r) between the average E and average thermal flux in the early morning to midday was r = -0.929 and from midday to midnight, it was r = 0.846 during the summer season (data not shown). Noteworthy is the fact that the cluster roots of the unfertilised plants grew in the 10−30 cm soil depth. In addition, the soil water content from the Diviner apparatus in the 20−30 cm soil layer was the highest compared to surface and deeper down the soil at the deep site during 2016/17 (Fig. 3). Theoretically, moisture in the rocky subsoil can move upwards due to temperature or water gradient and condense in the drying front, either on the cluster roots or on the soil particles when the daily soil temperature is at its lowest. The cluster roots may have the ability to take up whatever small quantities of water that condenses and enables the rooibos plant to survive during the annual summer drought. In fact, the fine root cluster of rooibos and the existence of a drying front at the same depth may not be a coincidence, but a specific adaptation of the root system to harvest small amounts of condensed water.

A smaller root system yielded a smaller above ground vegetative growth than a larger root system did. The results in the current study indicate that the ability of rooibos plants to produce AGB on shallow soils was much reduced by a lack of soil depth under rain-fed conditions. The low AGB and BGB of the fertilised treatment at both sites were probably due to the high P concentration in the soil since high P concentration can inhibit the cluster root growth of rooibos. The results of the taproots clearly demonstrate the benefits of having a deeper soil, rather than shallow soil for the production of rooibos plants in terms of the taproot penetration into the soil. Furthermore, this finding is according to expectation since the soil texture was homogenous and had no compaction that could have restricted root penetration. The taproots on the shallow soils were short and distorted (Fig. 8) due to restriction of the hard rock below 40 cm in the soil. Similar results were reported by Ricahrds^68^ where the taproot of *Protea compacta* (fynbos plant) extended from 0.4–0.6 m and 1.0 m in shallow and deep soils, respectively.

## Conclusion

The current study proved that soil depth is a critical factor in the production of rain-fed rooibos in its native low-rainfall habitat, particularly during droughts. The deeper soil allowed deeper root growth with longer taproots and also stored more rainwater, all of which are important production and survival factors under dryland conditions. These advantages of deep soils resulted indeed in significantly more aboveground growth compared to the shallow soil treatment. Irrespective of different soil depths, however, the rooibos test plants maintained a constant root to shoot ratio of ~ 0.3 which is indicative of the plant’s ability to balance its above-ground and below-ground growth.

In contrast to the positive effect of a deep soil, fertilisation caused a chain of negative responses. Above-ground as well as root growth of fertilised rooibos plants decreased, confirming the findings of other studies, namely that especially too high a P content of the soil can be toxic to rooibos. This drastically reduced growth, however, only led to marginally lower ΣET over the season compared to unfertilised plots. This unexpected high ΣET on fertilised plots was ascribed to the indirect effect of less ground cover and more direct sunlight on the soil surface as confirmed by significantly higher soil temperatures.

The water dynamics of bare soil was disappointingly similar to those of the soils planted to rooibos and its E closely followed the values of the other treatments. Furthermore, the rainless summer fallow period was too long and hot to store rainwater from the previous winter and spring rains until the next winter when rooibos is normally planted. At the end of the season in April, the SWC of the bare soil, was comparable and well below PWP, prove that fallowing (water harvesting) is not an option for the successful establishment of rooibos plants on the sandy soils of this dry region.

Evapotranspiration of rooibos was higher during the winter when some rain fell than during the hot dry summer months irrespective of treatment applied. This reversed ET pattern can be ascribed to the very low SWC during summer and the subsequent low evaporation. The rooibos plants survived under those conditions either through its specially adapted root system or through, yet unexplained, redistribution of small quantities of water in the soil. Based on diffusivity measurements, it is suggested that the drying-wetting (condensation/re-evaporation) cycle in the drying front can be a further important factor in the survival of rooibos plants during the annual summer droughts. These preliminary findings should be re-examined in further investigations to determine the validity and extent of the drying-wetting cycle theory.

## Data Availability

The datasets used and/or analysed during the current study available from the corresponding author on reasonable request.
